# A Rare Case of Spontaneous Pneumocephalus Associated with Nontraumatic Cerebrospinal Fluid Leak

**DOI:** 10.1155/2016/1828461

**Published:** 2016-04-27

**Authors:** Murad Baba, Omer Tarar, Amer Syed

**Affiliations:** Department of Internal Medicine, Jersey City Medical Center, 355 Grand Street, Jersey City, NJ 07302, USA

## Abstract

*Introduction*. Spontaneous nontraumatic pneumocephalus (PNC) and cerebrospinal fluid (CSF) leaks are both very uncommon conditions. We report a rare case of spontaneous pneumocephalus associated with CSF leak secondary to right sphenoid sinus bony defect without history of trauma.* Case Description*. 51-year-old Hispanic female with past medical history of hypertension and idiopathic intracranial hypertension (Pseudotumor Cerebri) presented to the emergency room complaining of headache and clear discharge from the right nostril. Physical examination was significant for right frontal sinus tenderness and clear discharge from right nostril. Computed Tomography (CT) scan of the brain showed moderate amount of extra-axial air within the right cerebral hemisphere indicative of pneumocephalus. CT scan of facial bones showed bony defect along the right sphenoid sinus with abnormal CSF collection. The patient was started on intravenous antibiotics for meningitis prophylaxis and subsequently underwent transsphenoidal repair of cerebrospinal fluid leak with abdominal fat graft. CSF rhinorrhea stopped completely after the surgery with near complete resolution of pneumocephalus before discharge.* Conclusions*. Early identification of pneumocephalus and surgical intervention can help decrease the morbidity and avoid possible complications. Idiopathic intracranial hypertension, although rare, can lead to CSF leak and pneumocepahlus.

## 1. Introduction

Pneumocephalus (PNC) is defined as pathological collection of gas within the cranial cavity accumulating in the epidural, subdural, subarachnoid, intraventricular, or intraparenchymal compartments [1]. The main cause of PNC is head injury, trauma accounts for 74% of all cases followed by intracranial neoplasms, infections, neurosurgery, paranasal sinus surgery, and diagnostic or neurosurgical interventions such as pneumoencephalography or lumbar puncture [2, 3].

Chiari was the first to describe PNC in 1884 [4]. The developmental mechanism of pneumocephalus is mainly based on two factors: a reduction in intracranial pressure and the presence of a defect in the dura. It is caused by either a ball-valve mechanism that allows air to enter but not to exit or by CSF leakage which creates a negative pressure with subsequent air entry [2].

## 2. Case Description

51-year-old Hispanic female with past medical history of hypertension and idiopathic intracranial hypertension (Pseudotumor Cerebri) presented to the emergency room complaining of headache for three weeks. Her headache was progressively worsening more on the right side, associated with clear discharge from right nostril which was aggravated by bending forward and straining. She denied any history of trauma. She reported having flu with frequent sneezing episodes around the same time. She reported similar symptoms six years ago; at that time further workup was done including Computed Tomography (CT) scan of the brain which was unremarkable and lumbar puncture (LP) which showed high opening pressure. She was diagnosed with idiopathic intracranial hypertension and treated conservatively.

**Figure 1:**
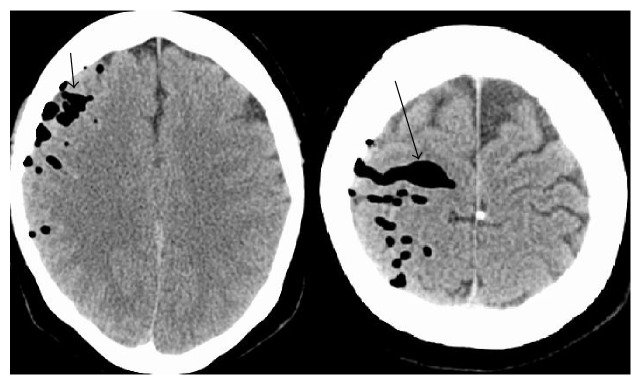
Axial Computed Tomography images of the brain showing air within the right cerebral hemisphere (arrows).

**Figure 2:**
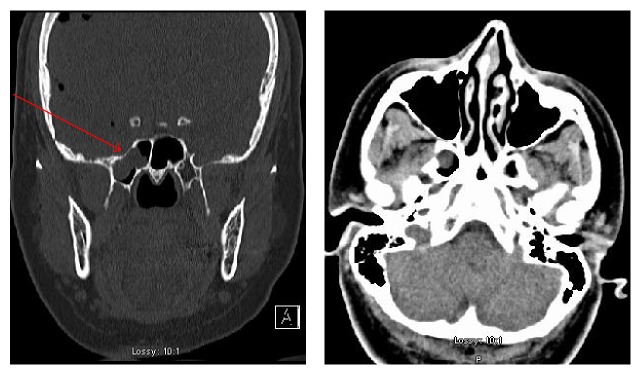
Axial and coronal Computed Tomography images of the facial bones showing right sphenoid sinus bony defect and CSF collection.

Physical examination was significant for right frontal sinus tenderness and clear discharge from right nostril. Initial laboratory workup did not reveal any significant results. CT scan of the brain showed moderate amount of extra-axial air within the right cerebral hemisphere indicative of pneumocephalus as shown in Figure 1. CT scan of facial bones showed focal bony defect along thin roof of right sphenoid sinus with abnormal CSF collection immediately above and within the lateral recess of the right sphenoid sinus (Figure 2). Metastatic disease was ruled out with a negative bone scan and unremarkable CT scans of the chest, abdomen, and pelvis.

The patient was started on intravenous antibiotics for meningitis prophylaxis with Ceftriaxone and Vancomycin, and the neurosurgery team decided to take her to the Operating Room (OR) where they did a lumbar drain and injected a Fluorescein dye which subsequently was visualized in the right sphenoid sinus during the operation. A CT cisternography was replaced with Fluorescein dye injection through the lumbar drain. She underwent transsphenoidal repair of cerebrospinal fluid leak with abdominal fat graft and lumbar drain placement.

CSF rhinorrhea stopped completely after the surgery, and the lumbar drain was removed after six days. Prior to discharge, a repeat brain CT scan was obtained and showed near complete resolution of the right hemispheric pneumocephalus. On further follow-up, she was seen in the clinic at one month and five months after surgery and her symptoms significantly improved.

## 3. Discussion

PNC is associated with several etiological factors, including head injury, surgical procedures, infection, and neoplasm. PNC is particularly frequent after head injury with concomitant skull base fractures and CSF leakage. Spontaneous, nontraumatic pneumocephalus is very uncommon, and most cases result from nose blowing, sneezing, and the Valsalva maneuver and by environmental pressures including those encountered in mountain climbing, during flights, and during scuba diving [2].

The presenting symptom in pneumocephalus is usually headache, and other symptoms include CSF rhinorrhea (as in our patient), meningeal signs, hemiparesis, papilledema, and cranial nerve palsies. However, the presentation of pneumocephalus is often vague.

Idiopathic intracranial hypertension most commonly affects women of child bearing age and is usually a self-limiting condition, and the association between idiopathic intracranial hypertension and spontaneous CSF rhinorrhoea is rare [5].

Persistent or tension PNC may cause headache, lethargy, and neurological deterioration. It reflects an abnormal communication between the intradural space and external environment, creating a risk factor for central nervous system infection [1]. The Mount Fuji sign on CT scans of the brain is a characteristic radiological finding defined as compression of frontal lobes and widening of the interhemispheric space between the tips of the frontal lobes; it is useful in discriminating tension pneumocephalus from nontension pneumocephalus.

Tension pneumocephalus occurs most commonly after the neurosurgical evacuation of a subdural hematoma; it can be a neurosurgical emergency. The prevalence of tension pneumocephalus following the evacuation of chronic subdural hematomas has been reported from 2.5% to 16%. Tension pneumocephalus can also occur as a result of skull base surgery, paranasal sinus surgery, posterior fossa surgery in the sitting position, or head trauma [6, 7].

Treatment of PNC is either conservative or surgical. This should be decided based on the type, severity, and presence of increased intracranial pressure. PNC has been treated by ventilating the patient with normobaric 100% oxygen, resulting in the reabsorption of nitrogen into the blood stream and reduction of the volume of the intracranial air [1, 8]. When tension PNC is suspected by clinical and imaging findings, treatment consists of emergent decompression to alleviate pressure on the brain parenchyma, as air is toxic to neurons and can cause further damage to the already compromised parenchyma, and that leads to cerebral edema surrounding the air evolving into encephalomalacia [9].

Surgical options for tension pneumocephalus include drilling of burr holes, craniotomy, needle aspiration, ventriculostomy placement, and closure of dural defect. In addition, patient education to avoid Valsalva's maneuvers or nose blowing can possibly contribute to reducing recurrence.

## 4. Conclusion

Few cases were described in the literature and most were treated surgically. Our rare case presented with nontraumatic CSF leak and spontaneous pneumocephalus; early identification of such cases and surgical intervention can help decrease the morbidity and the complications that can happen.

CSF rhinorrhoea is a rare complication of idiopathic intracranial hypertension; if it happens, it can lead to meningitis and PNC; therefore surgical repair is always recommended.

## Abbreviations

PNC: Pneumocephalus
CSF: Cerebrospinal fluid
CT: Computed Tomography
LP: Lumbar puncture.
